# Comparison of Recurrence Rates of Odontogenic Keratocyst and Ameloblastoma following surgical excision and peripheral ostectomy in the Maxilla

**DOI:** 10.1186/s40902-025-00488-3

**Published:** 2025-10-21

**Authors:** Young Heon Jeong, Jin Seok Kim, Heonwoo Lee, Kang-Min Ahn

**Affiliations:** 1 Department of Oral and Maxillofacial Surgery, University of Ulsan, Seoul, Korea, Republic of; 2 Department of Pathology, University of Ulsan, Seoul, Korea, Republic of

**Keywords:** Odontogenic keratocyst, Ameloblastoma, Recurrence, Odontogenic tumors, Odontogenic cysts, Maxilla

## Abstract

**Background:**

Odontogenic keratocyst (OKC) and ameloblastoma are benign but locally aggressive jaw lesions that require careful surgical management. While radical resection has traditionally been the preferred treatment, conservative approaches such as surgical excision with peripheral ostectomy have gained interest to prevent significant functional and aesthetic consequences. This study aims to compare the recurrence rates of OKC and ameloblastoma in the maxilla following conservative surgical treatment.

**Methods:**

A retrospective analysis was performed on 31 patients who underwent surgical excision with peripheral ostectomy for OKC (*n* = 19) or ameloblastoma (*n* = 12) in the maxilla between 2006 and 2024. Recurrence was monitored through periodic radiographic follow-ups (mean duration: 43 months), including panoramic radiograph and cone-beam computed tomography (CBCT).

**Results:**

Among the 19 OKC cases, tumor recurred in two patients (10.5%) at 5, and 14 years post-surgery, while no recurrences were found in the ameloblastoma group. The majority of OKC (95%) were located in the posterior maxilla, whereas ameloblastoma showed an even distribution between the anterior and posterior regions, with 50% in each. Although Fisher’s Exact Test did not demonstrate a statistically significant difference in recurrence rates, simple comparison suggests that OKC may have a higher tendency for recurrence than ameloblastoma following conservative treatment.

**Conclusion:**

Surgical excision with peripheral ostectomy appears to be a viable conservative treatment option for both OKC and ameloblastoma in the maxilla, with a relatively low recurrence rate observed in this study. Notably, recurrences observed even after long-term follow-up indicate the necessity for prolonged monitoring.

## Background

Ameloblastoma is a benign odontogenic tumor derived from odontogenic epithelium, such as rests of dental lamina, rest of Malassez, reduced enamel epithelium, or basement membrane of oral mucosa [1]. It accounts for 14% of all jaw tumors and cyst, considered as the most prevalent odontogenic tumors [2]. Although it is classified as a benign by The 2022 World Health Organization (WHO) classification, it shows locally aggressive growth pattern and has high recurrence rates [2]. Up to 3.4% of ameloblastoma cases undergo malignant transformation, and up to 2% of them metastasize to other sites [3, 4](Liu ~,2025). Histologically, ameloblastoma can be classified into solid/multicystic, unicystic, and peripheral variants, with the solid/multi-cystic type demonstrating the highest recurrence rate [1, 5]. Historically, radical surgical approaches such as segmental or marginal resection have been the treatment of choice, particularly for solid/multicystic ameloblastoma [6]. However, radical excision is associated with significant morbidity, including facial deformity and functional impairment, prompting a shift toward more conservative techniques in select cases [7]. Among these, surgical excision combined with peripheral ostectomy has emerged as a potential alternative, aiming to remove residual tumor cells while preserving adjacent structures [8]. Ameloblastoma most commonly arises in the mandible (80%), but maxillary involvement occurs in approximately 20% of cases, often presenting a greater surgical challenge due to its proximity to anatomical structures such as the maxillary sinus, nasal cavity, orbit, and pterygoid plate [9].

Odontogenic keratocyst (OKC), derived from the rests of dental lamina, account for 3–11% of all odontogenic cysts and are characterized by an aggressive, locally invasive nature with a high recurrence rate [10, 11]. Unlike ameloblastoma, OKC tends to grow along the jaw’s long axis, resulting in less pronounced jaw swelling [12]. Multiple OKC exhibits a strong association with Gorlin-Goltz syndrome [13, 14]. OKC has a tendency for satellite cyst formation, which can contribute to recurrence even after enucleation [13]. OKC is more frequently found in the mandible, particularly in the posterior region, but maxillary cases present additional challenges due to thin cortical bone and proximity to the sinus [15]. OKC occurs less commonly in the maxilla, comprising approximately 22% of cases, with the maxillary sinus being the rarest site of involvement, accounting for only around 1% [16]. The surgical management of OKC remains controversial due to their high recurrence rates, ranging from 12 to 62%, depending on the treatment modality [17]. Enucleation alone has been associated with the highest recurrence risk, leading to the adoption of adjunctive procedures such as peripheral ostectomy, Carnoy’s solution application, or decompression to improve outcomes [18]. Peripheral ostectomy is particularly beneficial in maxillary cases where radical resection may cause significant morbidity, helping to eliminate residual cystic epithelium while preserving anatomical structures [19].

Given the slow-growing nature of both lesions and their relatively low metastatic potential, treatment strategies should not focus only for recurrence prevention but also consider functional and aesthetic outcomes [20]. Conservative surgical approaches, such as surgical excision with peripheral ostectomy, can minimize patient morbidity [21]. Since OKC and ameloblastoma tend to occur at a younger age compared to oral malignancies, the benefits of conservative surgical approaches, which preserve both function and aesthetics, become even more significant. OKC can develop across a wide age range, from the first to the ninth decades of life, with peak incidence in the third decade [22]. Similarly, ameloblastoma is most found between the ages of 30 and 60, with an average onset at 36 years and a peak occurrence in the fifth decade [1, 23]. In contrast, oral squamous cell carcinoma, the most common malignancy in oral cavity, predominantly affects older individuals, with most cases occurring between 65 and 84 years of age [24]. Given that patients with OKC and ameloblastoma is often younger and have a longer life expectancy, preserving anatomical structures and minimizing surgical morbidity through conservative approaches are more important for long-term function and aesthetics [21]. Previous studies have examined recurrence rates following various surgical methods. In the mandible, when the tumor or cyst extends inferiorly, peripheral ostectomy is often not feasible due to the risk of pathologic fracture, and segmental ostectomy is frequently required, making accurate comparison between the two lesions difficult. In contrast, in the maxilla, both ameloblastoma and OKC could be managed consistently with the same surgical method, providing a unique opportunity for direct comparison, yet studies directly comparing recurrence rates after surgical excision and peripheral ostectomy remains limited. By analyzing long-term outcomes of this approach, this study aims to determine whether conservative treatment can be an alternative to radical surgical interventions.

## Methods

This retrospective study included 31 patients diagnosed with OKC or ameloblastoma in the maxilla who underwent surgery performed by a single surgeon between 2006 and 2024. This retrospective study was approved by the institutional review board (IRB) in our hospital (IRB no. S2025-0347–0001) and was conducted in accordance with the Helsinki Declaration of 1975 as revised in 2000. All patients underwent surgical excision (or simple enucleation) with peripheral ostectomy (depth: 1–2 mm). Surgical excision was done using Molt curette and a surgical curette, with careful manipulation to avoid rupturing the cyst or tumor. Peripheral ostectomy was subsequently carried out using a low-speed handpiece with an oval or round bur. In cases where impacted or involved teeth were present, tooth extraction was performed. However, if the tumor involved the mucosa or extended into the maxillary sinus, no additional treatment was applied to the underlying mucosa following tumor removal. Postoperatively, patients were periodically monitored using panoramic radiographs or cone-beam computed tomography (CBCT) to assess recurrence. If recurrence was detected, re-operation was performed, followed by continued routine follow-up. In cases where tooth extraction was necessary during surgery, dental implant placement was avoided to prevent interference with radiographic evaluation of recurrence due to imaging artifacts. Instead, dentures were applied, or in cases involving the most posterior area, an edentulous state was maintained. In one case, however, the extent of the posterior tooth loss was substantial, and implant placement was performed after a 5-year follow-up period without recurrence. The location of tumor was classified into anterior or posterior regions, using the second premolar as the boundary. Given the small sample size, Fisher’s Exact Test was used for statistical analysis to compare recurrence rates between groups. The level of significance was set as *P* < 0.05. Statistical analyses were carried out using the IBM SPSS for Windows (ver. 21.0; IBM Corp., Armonk, NY, USA).

## Results

A total of 31 patients were included in this study, with 19 diagnosed with OKC and 12 with ameloblastoma in the maxilla. The OKC group comprised 5 males and 14 females, with a mean age of 39 years, while the ameloblastoma group consisted of 11 males and 1 female, with a mean age of 51 years. The average follow-up period was 2 years and 8 months for OKC patients and 2 years and 8 months for ameloblastoma patients (Table 1). Among the OKC cases, recurrence was observed in 2 patients (10.5%), occurring 4, and 14 years post-surgery at the same site. In contrast, no recurrences were observed in the ameloblastoma group during the follow-up period. However, it was not statistically significantly different through Fisher’s exact test (Table 1).

**Table 1:** Patients’ demographics, follow-up period, recurrence rate

Group	Total Patients	Male	Female	Mean Age (years)	Follow-up Period				Recurrence (recurrence rate)
					Average (months)	< 2 years	2–5 years	> 5 years	
OKC	19	5	14	38	49.2	7	6	6	2 (10.5%)
Ameloblastoma	12	11	1	51	32.5	5	5	2	0 (0%)
Total	31	16	15	43	42.8	12	11	8	

^*^ Recurrence of OKC was observed in 3 patients, occurring 5, and 14 years post-surgery at the same site

^*^ Fisher’s exact test; *p* value = 0.244, indicating that difference in recurrence rates of OKC and ameloblastoma is not statistically significant. (significance level, *p* = 0.05)

^**^ *OKC* Odontogenic keratocyst

OKC lesions were predominantly located in the posterior maxilla, with 18 out of 19 cases (95%) occurring posterior to the second premolar, and only 1 case (5%) in the anterior region. In contrast, ameloblastoma was more evenly distributed, with 6 cases (50%) in the anterior maxilla and 6 cases (50%) in the posterior maxilla. In this study, the posterior boundary of the anterior area was set to the second premolar for classification. Fisher’s exact test for anatomical distribution of OKC and ameloblastoma was performed. It suggests that the difference in anatomical distribution is statistically significant (Table 2). Adherence to the sinus mucosa occurred in 17 of 19 OKC cases (89%) and in 7 of 12 ameloblastoma cases (58%) (Table 2).

**Table 2:** Location of tumor (anterior and posterior area)

Group	Anterior area	Posterior area	Total	adherence to the sinus mucosa
OKC	1 (5%)	18 (95%)	19 (100%)	17 (89%)
Ameloblastoma	6 (50%)	6 (50%)	12 (100%)	7 (58%)

^*^ Boundary of anterior and posterior area was set by second premolar of maxilla

^*^ Fisher’s exact test; *p* value = 0.007, indicating that difference in anatomical distribution of OKC and ameloblastoma is statistically significant. (significance level, *p* = 0.05)

^**^OKC Odontogenic keratocyst

In the OKC group, 3 patients (16%) had lesions smaller than 2 cm, 11 patients (58%) measured between 2 and 4 cm, and 5 patients (26%) larger than 4 cm, whereas in the ameloblastoma group, 3 patients (25%) having tumors smaller than 2 cm, 2 patients (17%) between 2 and 4 cm, and 7 patients (58%) larger than 4 cm (Table 3).

**Table 3:** Tumor size distribution

Group	< 2 cm	2–4 cm	> 4 cm	Total
OKC	3 (16%)	11 (58%)	5 (26%)	19 (100%)
Ameloblastoma	3 (25%)	2 (17%)	7 (58%)	12 (100%)

^**^OKC Odontogenic keratocyst

### Case 1

**Fig. 1:**
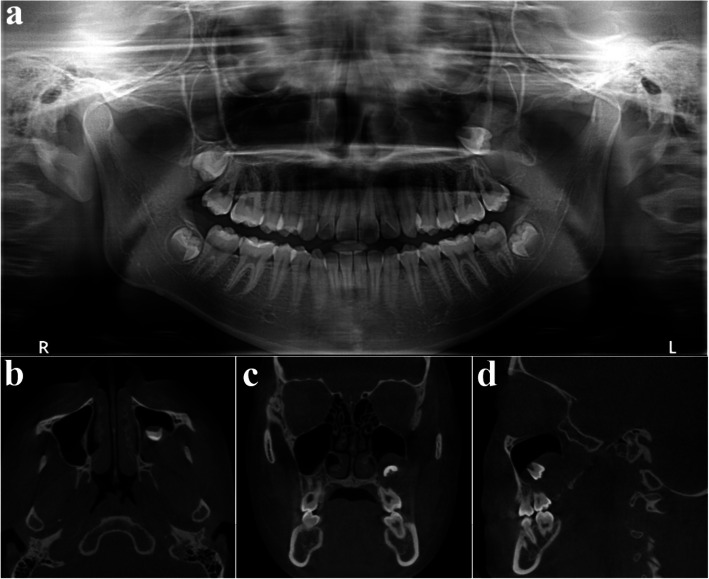
Preoperative radiographic images of a 15-year-old female patient with odontogenic keratocyst (OKC) involving an impacted left upper third molar, (**a**) panoramic radiograph, (**b**) CBCT, axial view, (**c**) CBCT, coronal view, (**d**) CBCT, sagittal view

**Fig. 2:**
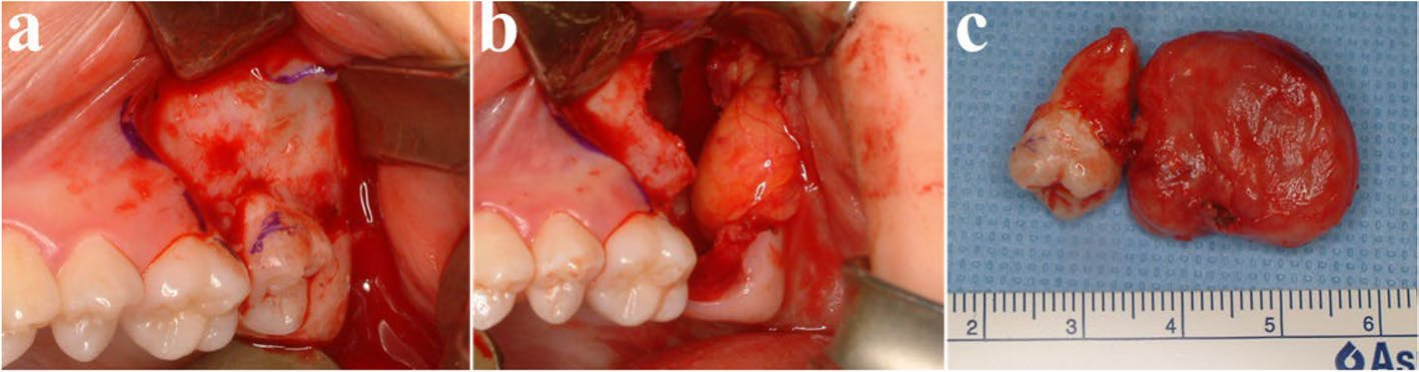
Surgical excision and peripheral ostectomy of OKC and specimen, (**a**) flap elevation was done, thinning and expansion of the cortical bone was observed, (**b**) surgical excision and peripheral ostectomy was done, buccal fat pad graft was performed to prevent oroantral fistula formation, (**c**) specimen with impacted third molar

**Fig. 3:**
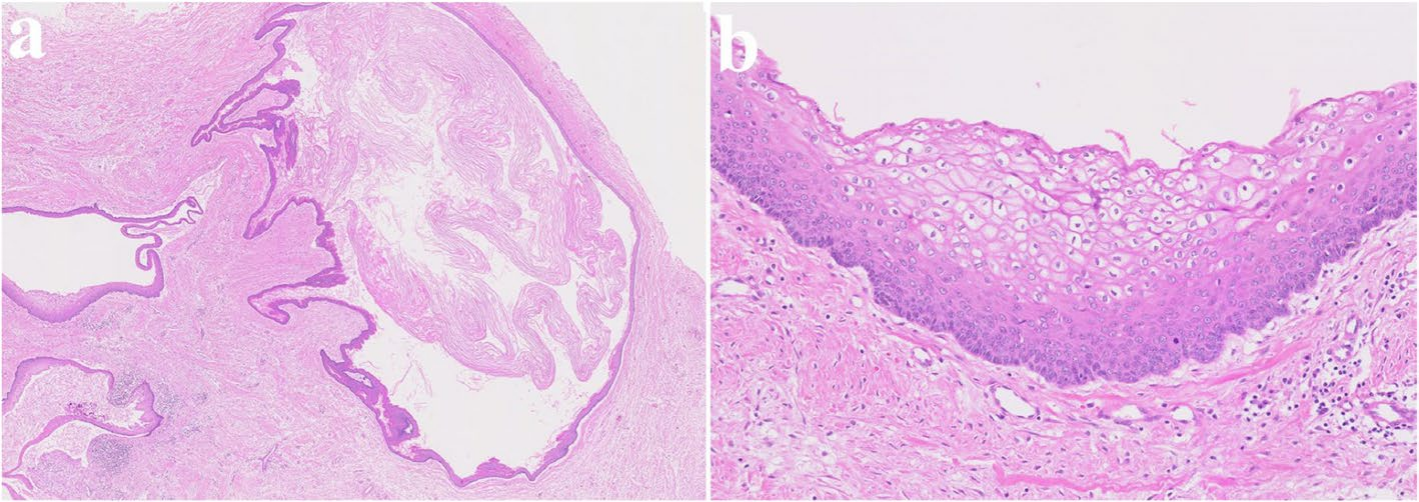
Histopathological slide of the lesion. **a** (× 2), showing multiple cysts. **b** (× 20) showing a thin epithelial lining

**Fig. 4:**
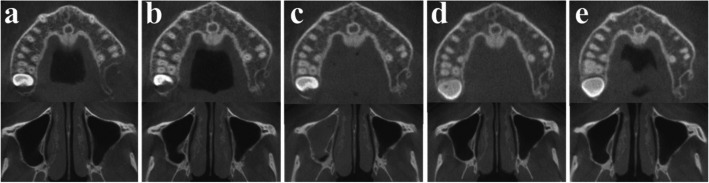
Regular follow-up of OKC after surgery using CBCT, (**a**) 3 months after surgery, (**b**) 1 year after surgery, (**c**) 2 years after surgery, (**d**) 3 years after surgery, (**e**) 5 years after surgery

A 23-year-old female was referred after a large cystic lesion was incidentally discovered on panoramic radiography at a local dental clinic (Fig. 1). CT imaging revealed a cystic lesion involving the left impacted maxillary third molar. With a provisional diagnosis of odontogenic keratocyst (OKC), surgical excision and peripheral ostectomy were performed under general anesthesia (Fig. 2). The cyst was removed without rupture including left maxillary third molar (Fig. 2d). The lesion was histologically diagnosed as an OKC (Fig. 3). The left maxillary second molar in contact with the cyst was also extracted. After the diagnosis of OKC, genetic testing for PTCH1 mutation was conducted and returned negative, thereby excluding Gorlin-Goltz syndrome and confirming the diagnosis of isolated OKC. The patient has been under follow-up for approximately 7 years, with no evidence of recurrence to date (Fig. 4).

### Case 2

**Fig. 5:**
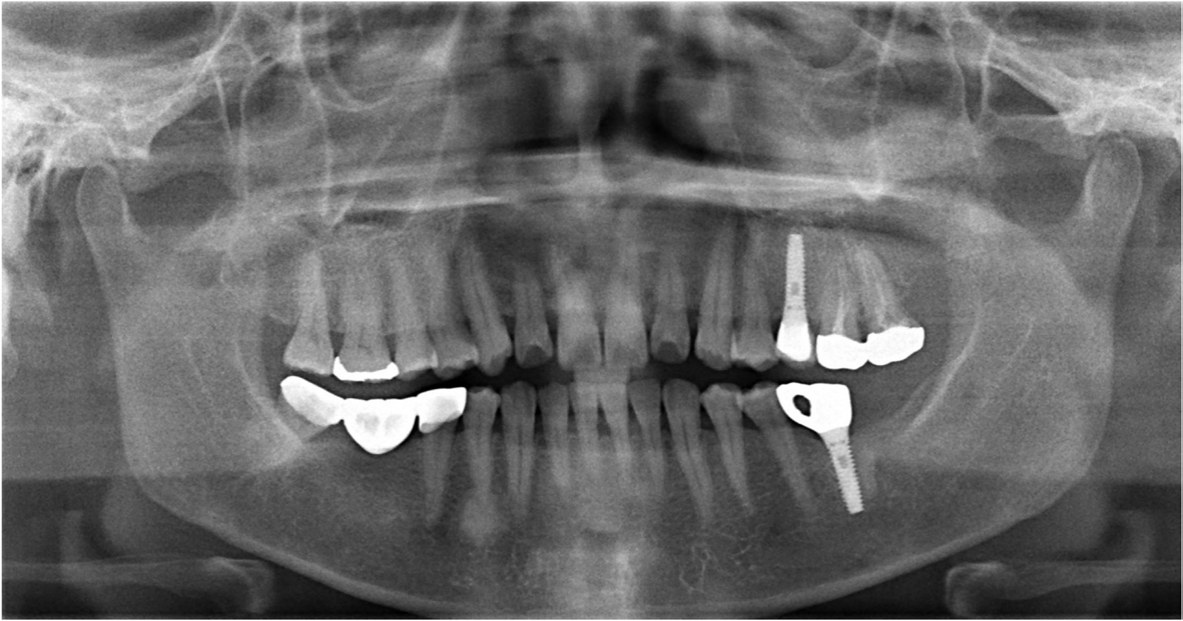
The panoramic radiograph of a 64-year-old female patient with odontogenic keratocyst (OKC) in left maxilla

**Fig. 6:**
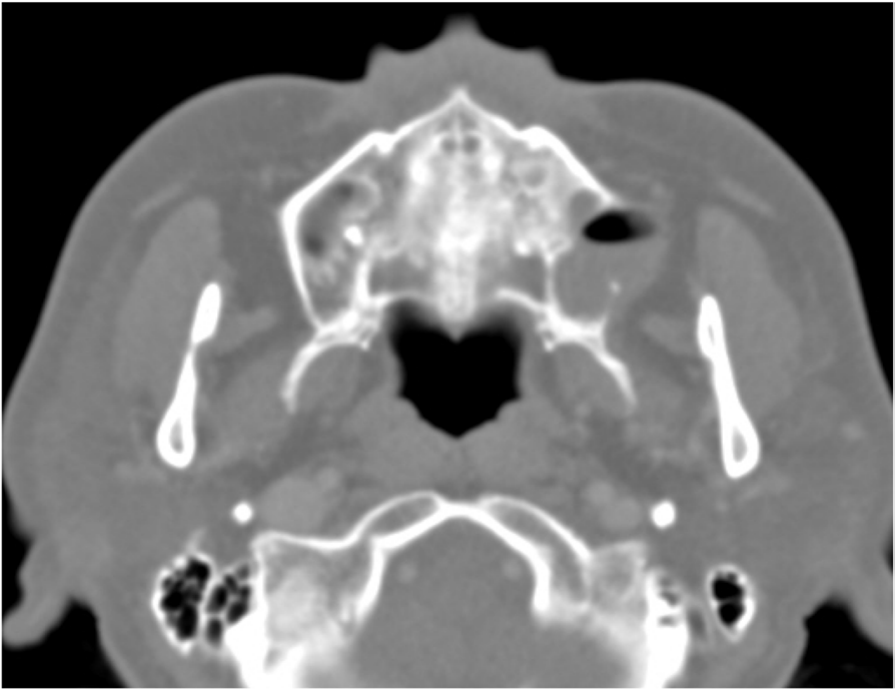
CT imaging showing a large cystic lesion invading the left maxillary sinus, with buccal cortical expansion and perforation

**Fig. 7:**
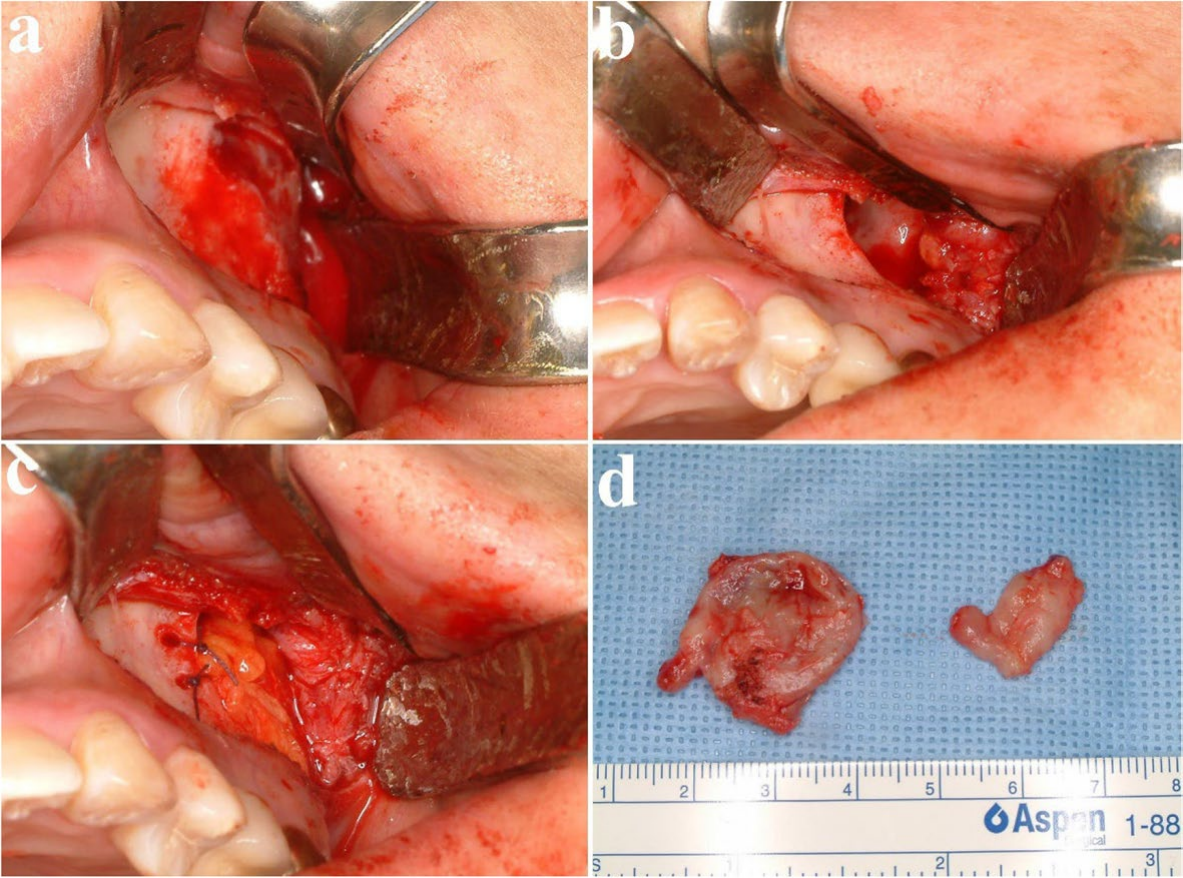
Surgical excision and peripheral ostectomy of first surgery. **a** flap elevation, showing cortical perforation (**b**) surgical excision and Peripheral ostectomy was done (**c**) Buccal fat pad graft was used to close the defect (**d**) specimen

A 64-year-old female presented with purulent discharge and intermittent dull pain in the left maxilla (Fig. 5). Her medical history included hypertension. Intraoral examination revealed a pus-like exudate and swelling in the buccal vestibule. CT imaging showed an extensive cystic lesion invading the maxillary sinus, with buccal cortical expansion and perforation (Fig. 6). Surgical excision and peripheral ostectomy were performed under general anesthesia, during which the cyst ruptured (Fig. 7). A buccal fat graft was done (Fig. 7c). No recurrence was noted on CT during regular follow-up for the first three years.

**Fig. 8:**
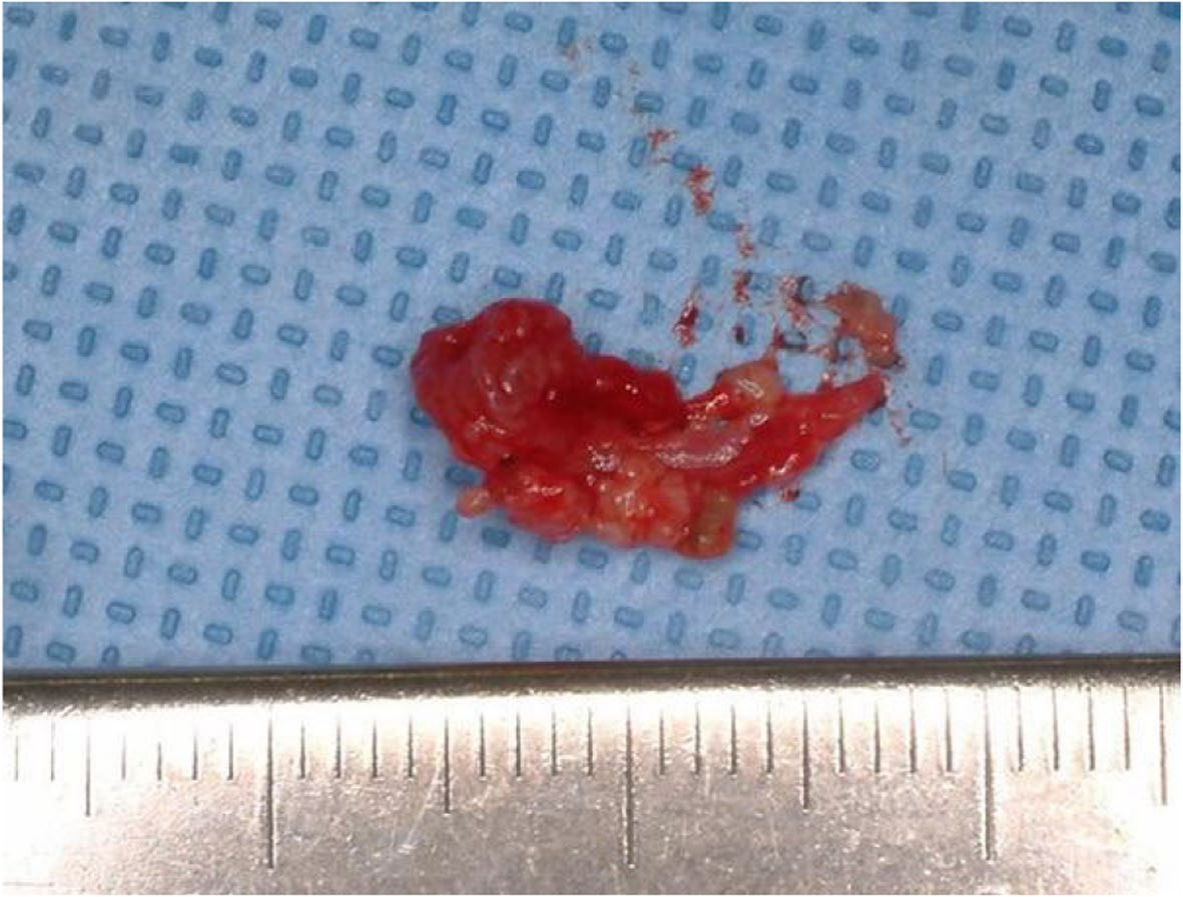
Second surgery, specimen

**Fig. 9:**
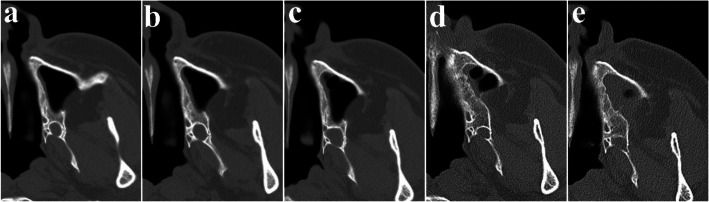
CT follow-up showing the slow growth rate of recurrent odontogenic keratocyst (OKC). **a** 3 years after the first surgery (maximum diameter: 7.76 mm), (**b**) 4 years after the first surgery (9.07 mm), (**c**) 5 years and 2 months after the first surgery (10.81 mm), (**d**) 3 years and 4 months after the second surgery (7.35 mm), (**e**) 4 years and 4 months after the second surgery (8.37 mm)

However, a cyst-like lesion with gradual enlargement was observed from the fourth year postoperatively, and a second surgery using the same method was performed five years after the initial procedure (Figs. 8 and 9a, b, c). A second recurrence was confirmed 3 years and 2 months after the second surgery, and reoperation is currently planned (Fig. 9d,e). Both recurrences occurred distal to the original lesion, adjacent to the pterygoid plate, with no associated impacted third molar (Fig. 9). Reoperation was postponed until definite lesion growth was observed, considering the typically slow growth rate characteristic of OKC (Fig. 9).

### Case 3

**Fig. 10:**
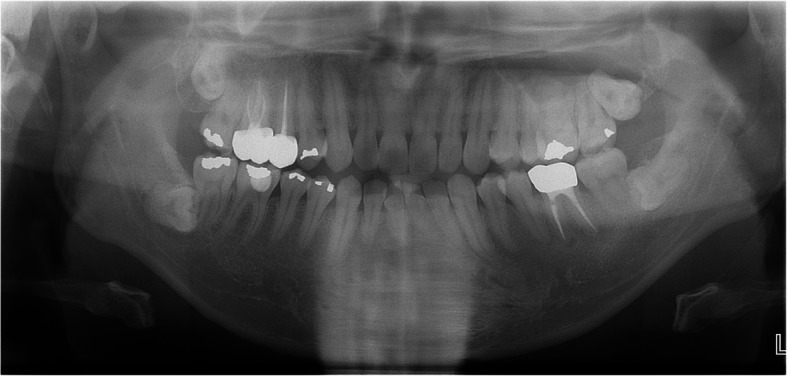
The panoramic radiograph of a 32-year-old male patient with odontogenic keratocyst (OKC) involving an impacted left upper third molar

**Fig. 11:**
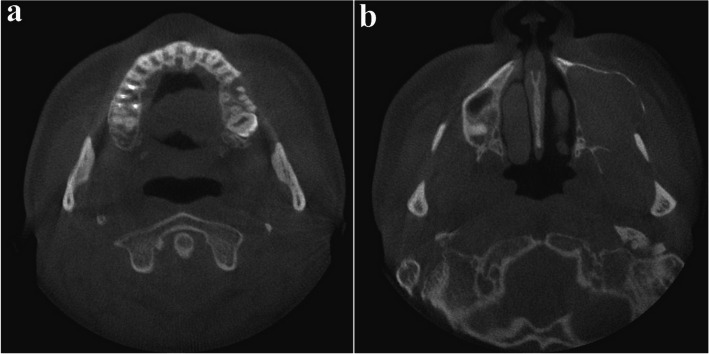
CT imaging, (**a**) showing radiolucent lesion involving left upper third molar, (**b**) showing large cystic lesion with buccal cortical expansion and perforation in left maxillary sinus

**Fig. 12:**
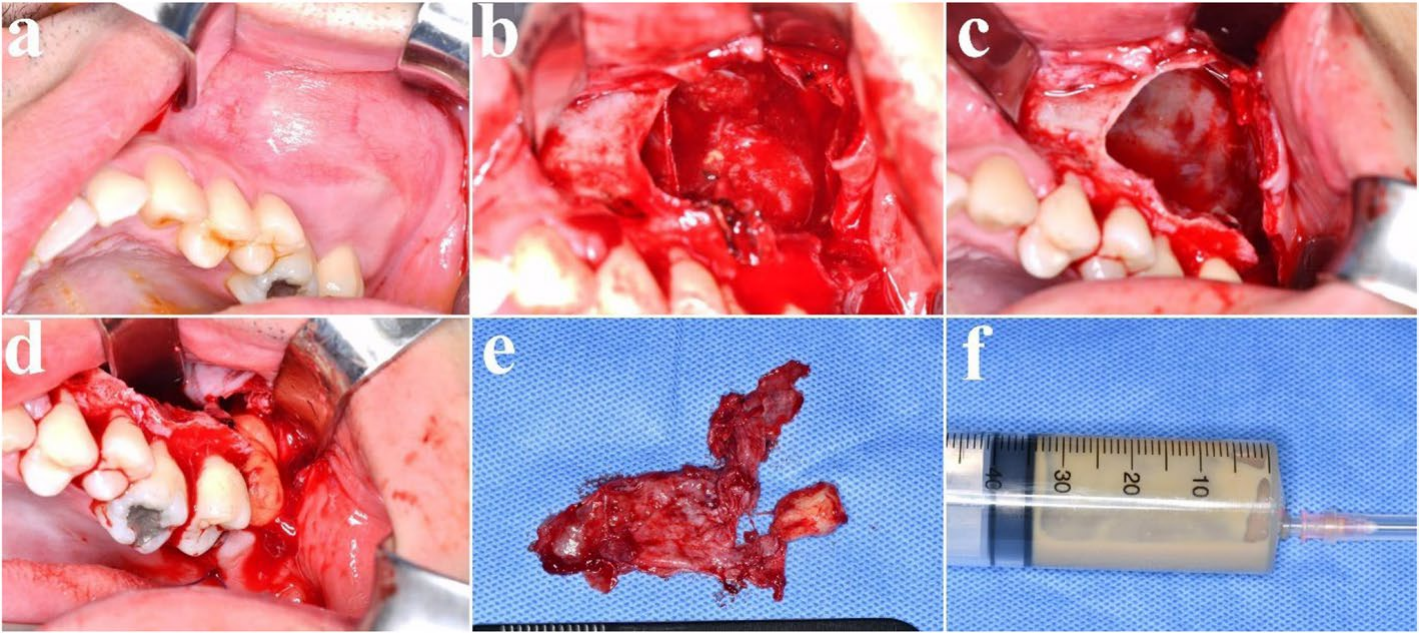
Surgical excision and peripheral ostectomy of OKC and specimen, (**a**) pre-operative photo, (**b**) flap elevation was done, cortical perforation and expansion of the cortical bone was observed, (**c**) surgical excision and peripheral ostectomy was done, (**d**) buccal fat pad graft was performed to prevent oroantral fistula formation, (**e**) specimen with impacted third molar, (**f**) Aspirated intracystic content of the OKC confirmed during surgery

**Fig. 13:**
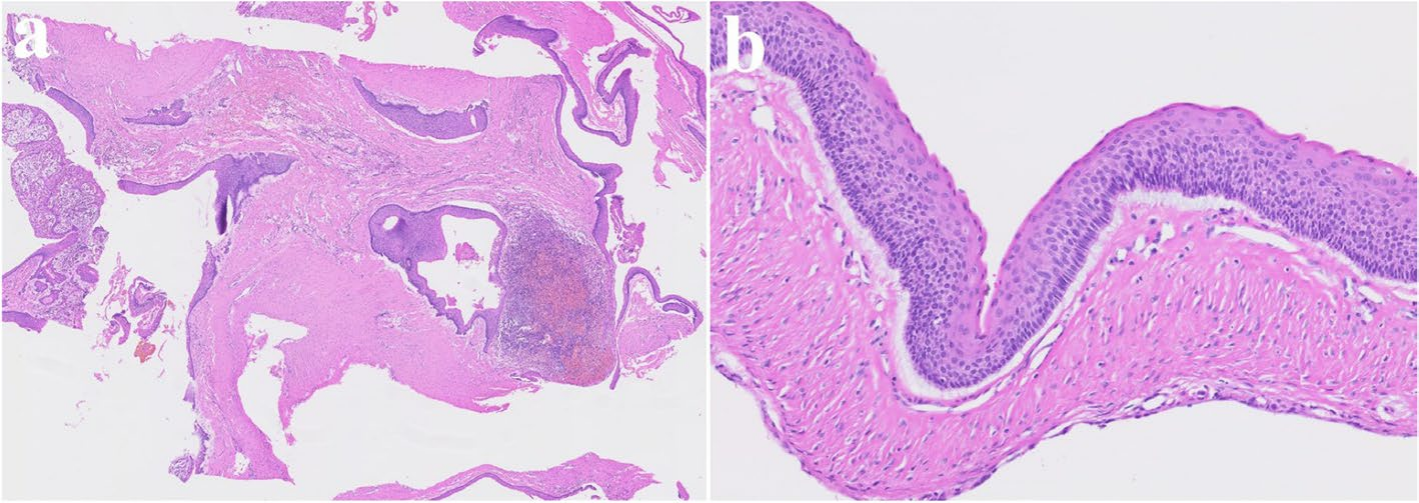
Histopathological slide of the lesion. **a** (× 2), showing multiple cysts and satellite cysts (**b**) (× 20) showing a thin epithelial lining

**Fig. 14:**
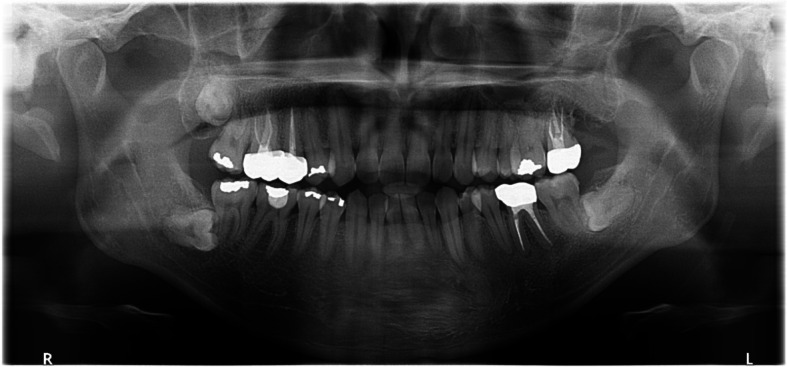
Panoramic radiograph at 1 year and 7 months postoperatively shows no evidence of recurrence

A 32-year-old male presented with a five-year history of swelling in the left maxilla (Fig. 10). CT imaging revealed a large cystic lesion involving the left maxillary third molar and completely occupying the maxillary sinus, with associated cortical expansion and perforation (Fig. 11). Surgical excision and peripheral ostectomy were performed under general anesthesia, including extraction of the involved third molar (Fig. 12). During surgery, aspiration suggested a diagnosis of OKC (Fig. 12f). A cyst measuring 5.5 × 3.5 × 0.3 cm was removed, although tearing occurred during the excision (Fig. 12e). Histopathological examination confirmed the diagnosis of OKC (Fig. 13). No evidence of recurrence has been observed during 1.5 years of follow-up (Fig. 14).

### Case 4

**Fig. 15:**
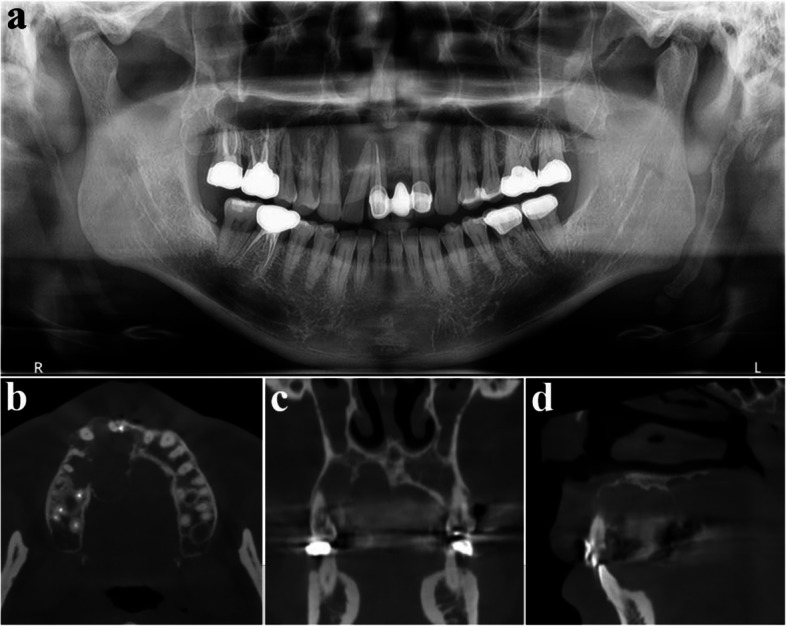
Preoperative radiographic images of a 41-year-old male patient with ameloblastoma in the maxillary anterior region, (**a**) panoramic radiograph, (**b**) CBCT, axial view, (**c**) CBCT, coronal view, (**d**) CBCT, sagittal view

**Fig. 16:**
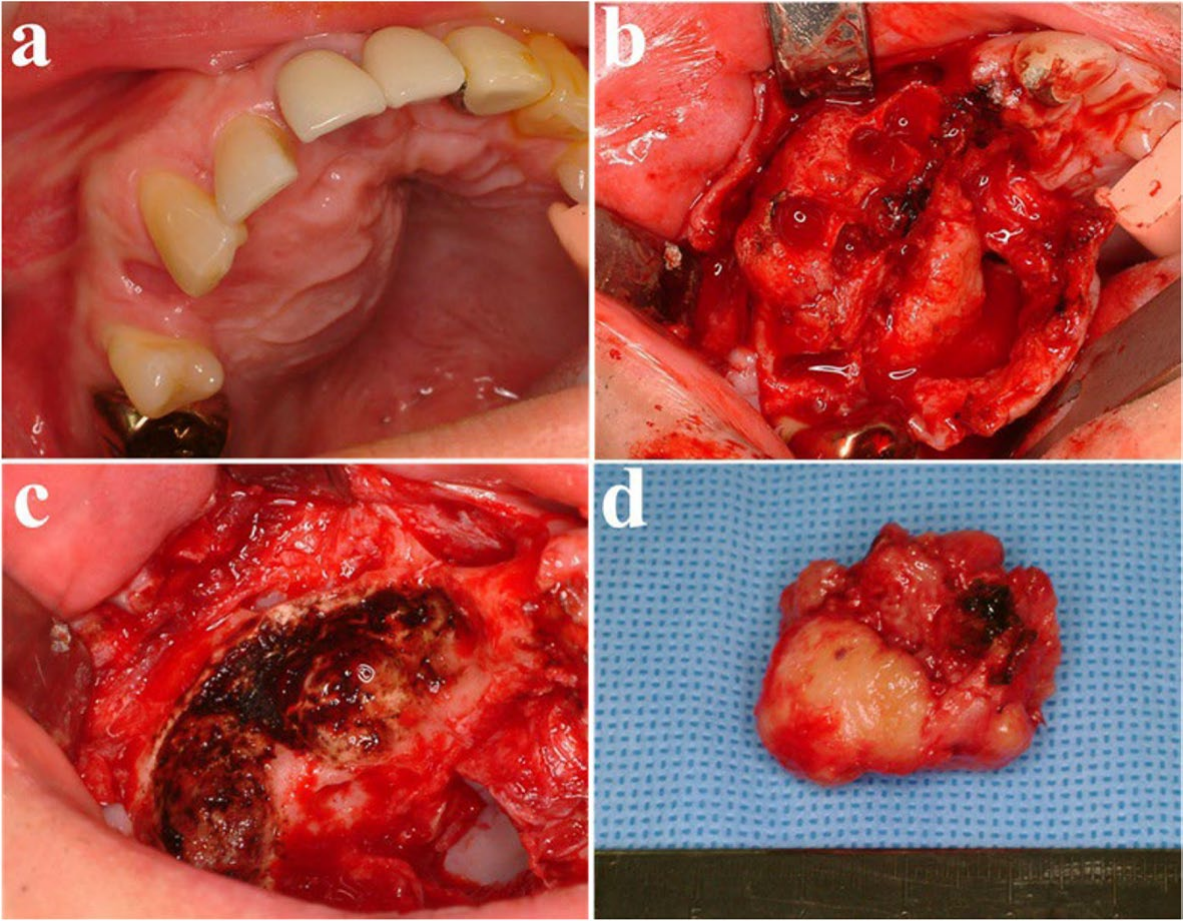
Surgical excision and peripheral ostectomy of ameloblastoma, (**a**) pre-operative photo, (**b**) flap elevation and extraction of multiple teeth were done, (**c**) surgical excision and peripheral ostectomy was done, (**d**) specimen

**Fig. 17:**
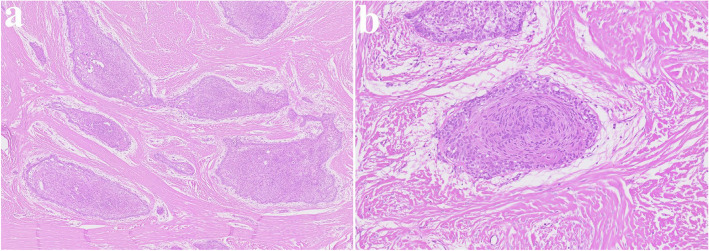
Histopathological slide of the lesion. **a** (× 5), Follicular type ameloblastoma **b** (× 20), peripheral palisading is seen around a stellate reticulum-like center

**Fig. 18:**
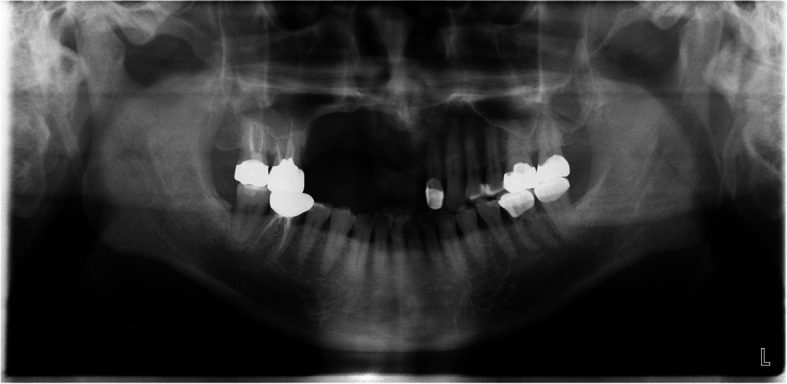
Panoramic radiograph at 2 years and 7 months postoperatively shows no evidence of recurrence

**Fig. 19:**
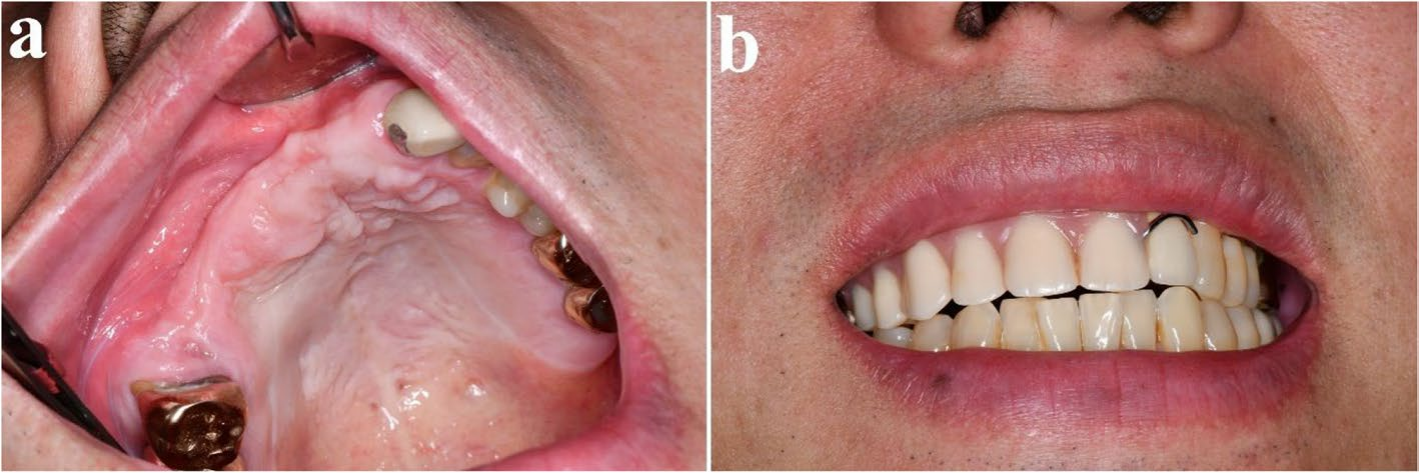
Clinical photos at 2 years and 7 months postoperatively

A 41-year-old male was referred from a local dental clinic due to a large radiolucent lesion in the right maxilla and swelling of palatal side (Figs. 15 and 16a). CT imaging showed a large, multilocular radiolucent lesion. Following an incisional biopsy, a diagnosis of ameloblastoma was confirmed. Surgical excision and peripheral ostectomy were performed, along with extraction of all involved teeth, including the right maxillary central incisor, lateral incisor, canine, and second premolar and the pathologic examination was performed (Figs. 16 and 17). A temporary denture was delivered starting one month postoperatively. The patient has been under regular follow-up for over two years, with no evidence of recurrence (Fig. 18). Although an alveolar bone defect remained, no aesthetic deficiency was observed when wearing the denture (Fig. 19).

### Case 5

**Fig. 20:**
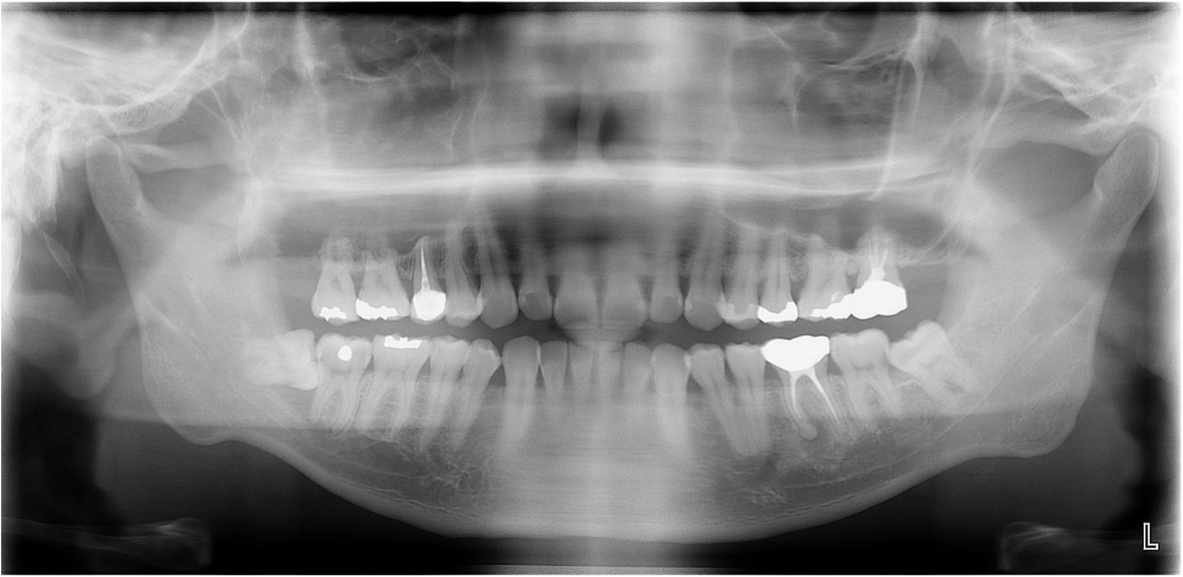
The panoramic radiograph of a 42-year-old male patient with ameloblastoma in the right maxilla

**Fig. 21:**
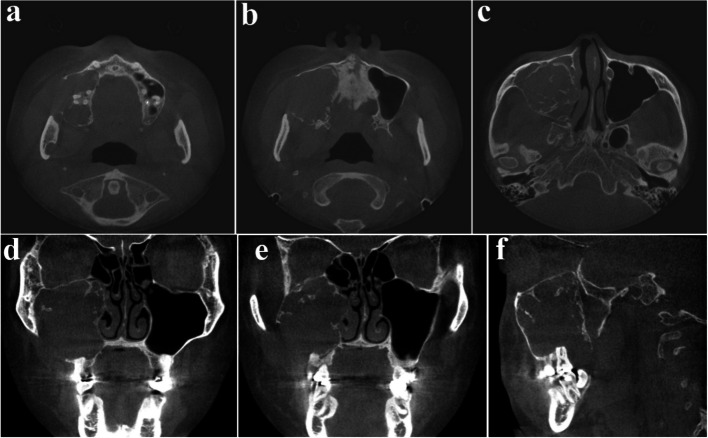
CT image showing extensive bone destruction from the maxillary alveolar ridge to the orbital floor with cortical expansion. **a** axial view, near the alveolar ridge, (**b**) axial view, maxillary sinus, (**c**) axial view, near the orbital floor (**d**) coronal view, premolar area, (**e**) coronal view, molar area, (**f**) sagittal view

**Fig. 22:**
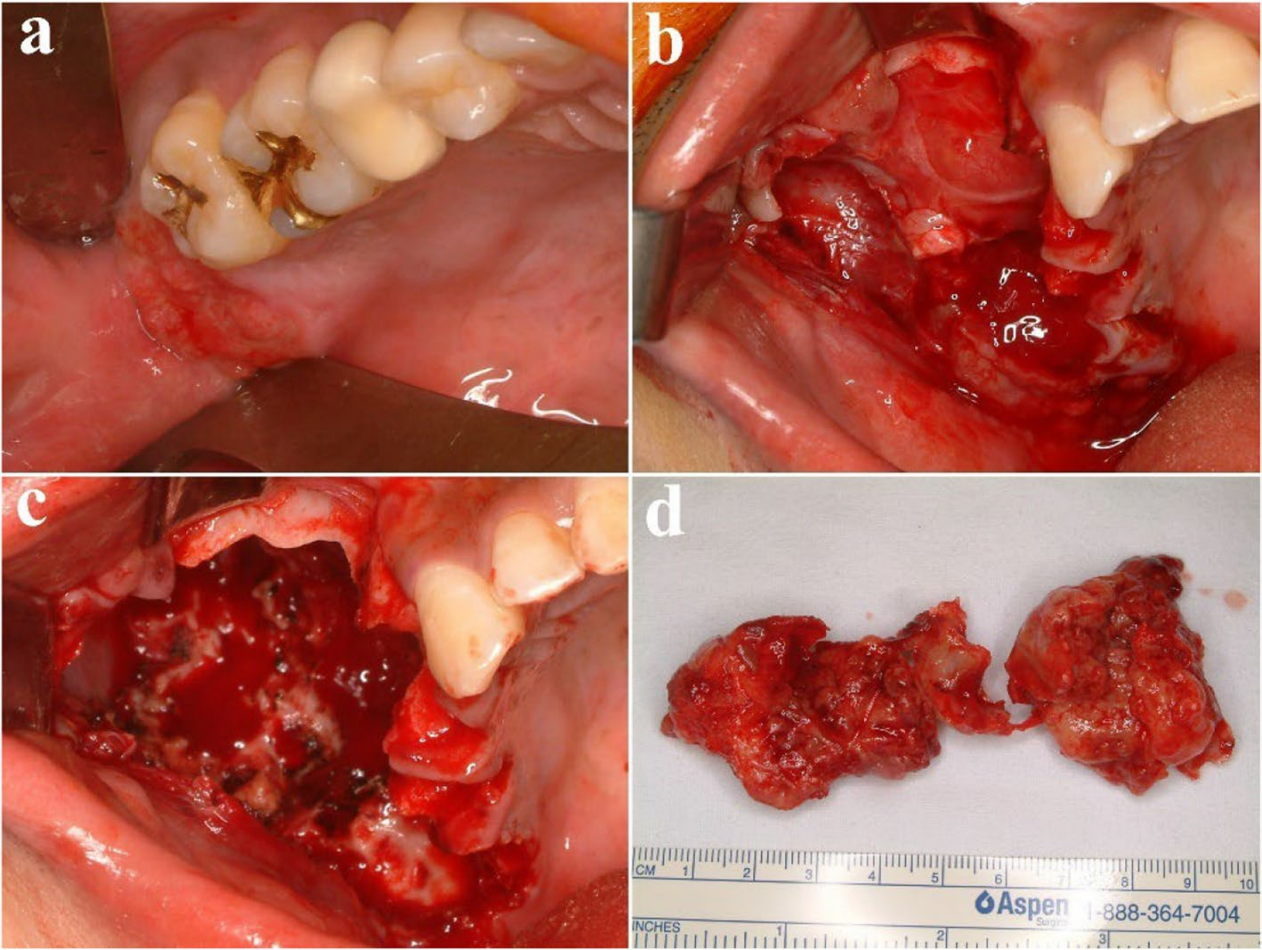
Surgical excision and peripheral ostectomy of ameloblastoma, (**a**) pre-operative photo, (**b**) flap elevation and extraction were done, (**c**) surgical excision and peripheral ostectomy was done, (**d**) specimen

**Fig. 23:**
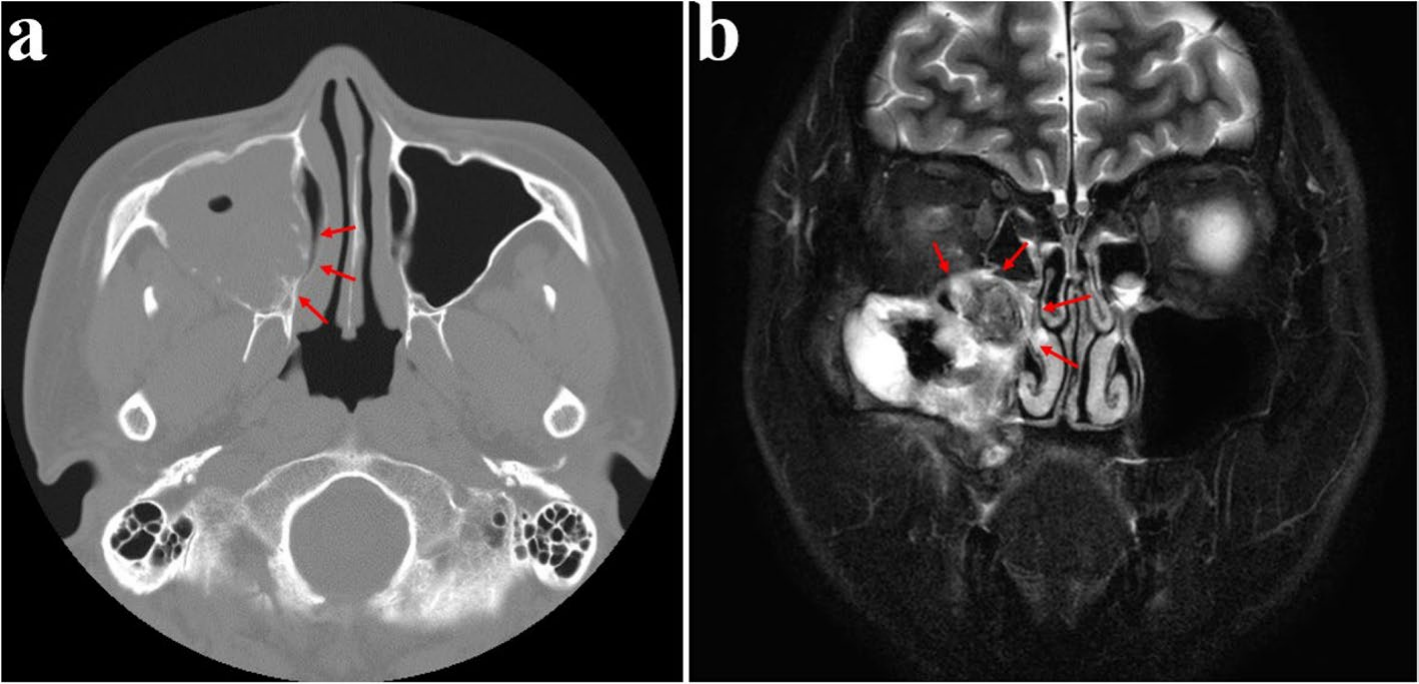
Four-month postoperative CT reveals residual tumor near the right ostium, (**a**) CT PNS (enhanced) axial view, (**b**) MR PNS (enhanced) coronal view

**Fig. 24:**
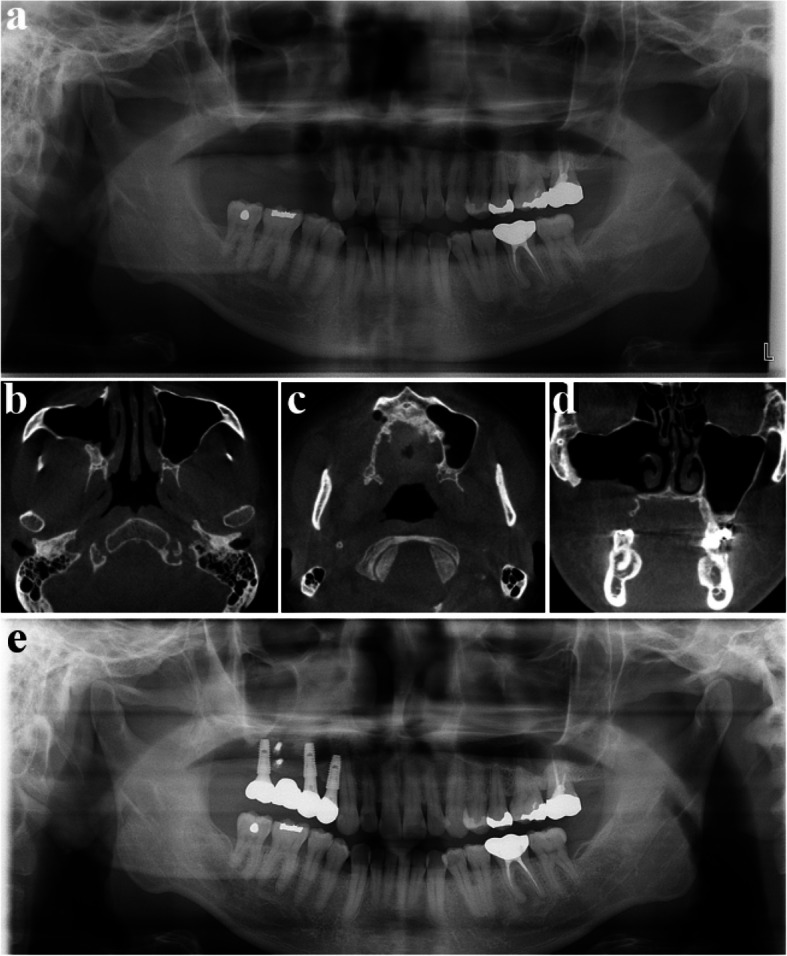
(**a**-**b**) No recurrence observed during over 5 years of follow-up, (**e**) After confirming a recurrence-free period of 5 years, ramal bone grafting and dental implant placement were performed to restore the defect. (7 years postoperative)

A 42-year-old male was referred for a large tumor in the right maxilla (Fig. 20). CT imaging revealed extensive bone destruction extending from the orbital floor to the maxillary alveolar ridge, with cortical expansion (Fig. 21). Initially, malignancy was suspected; however, histopathological examination after incisional biopsy confirmed the diagnosis of ameloblastoma. Surgical excision and peripheral ostectomy were done under general anesthesia, along with extraction of the right maxillary first and second premolars, and first and second molars (Fig. 22). A large tumor measuring 7.5 × 4.5 × 3.0 cm was removed (Fig. 22d). At 4 months postoperatively, a residual tumor near the ostium was identified on CT and was subsequently removed by the otolaryngology team (Fig. 23). No recurrence has been observed during over seven years of follow-up. After confirming a recurrence-free period of five years, ramal bone grafting and dental implant placement were performed to restore the defect (Fig. 24).

### Case 6

**Fig. 25:**
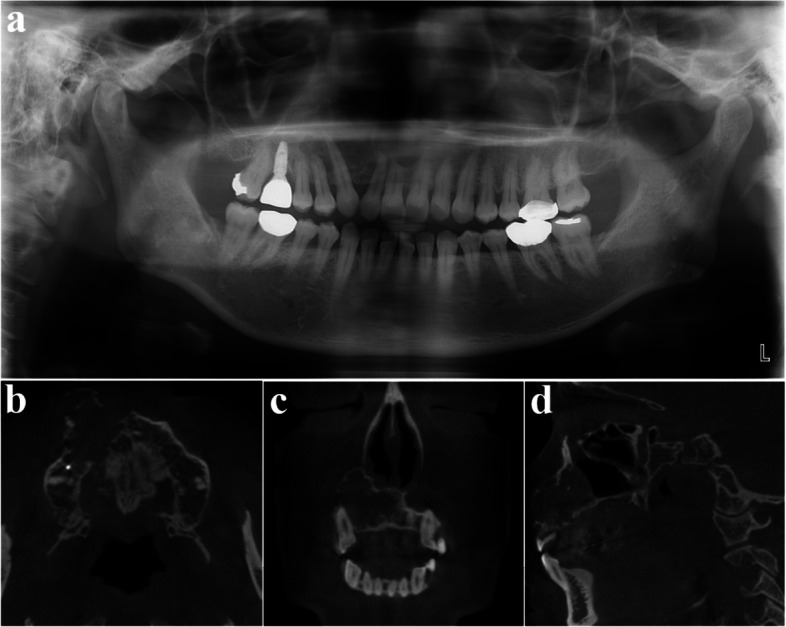
Preoperative radiographic images of a 53-year-old female patient with ameloblastoma in the right maxilla with displacement of multiple teeth, (**a**) panoramic radiograph, (**b**) CBCT, axial view, (**c**) CBCT, coronal view, (**d**) CBCT, sagittal view

**Fig. 26:**
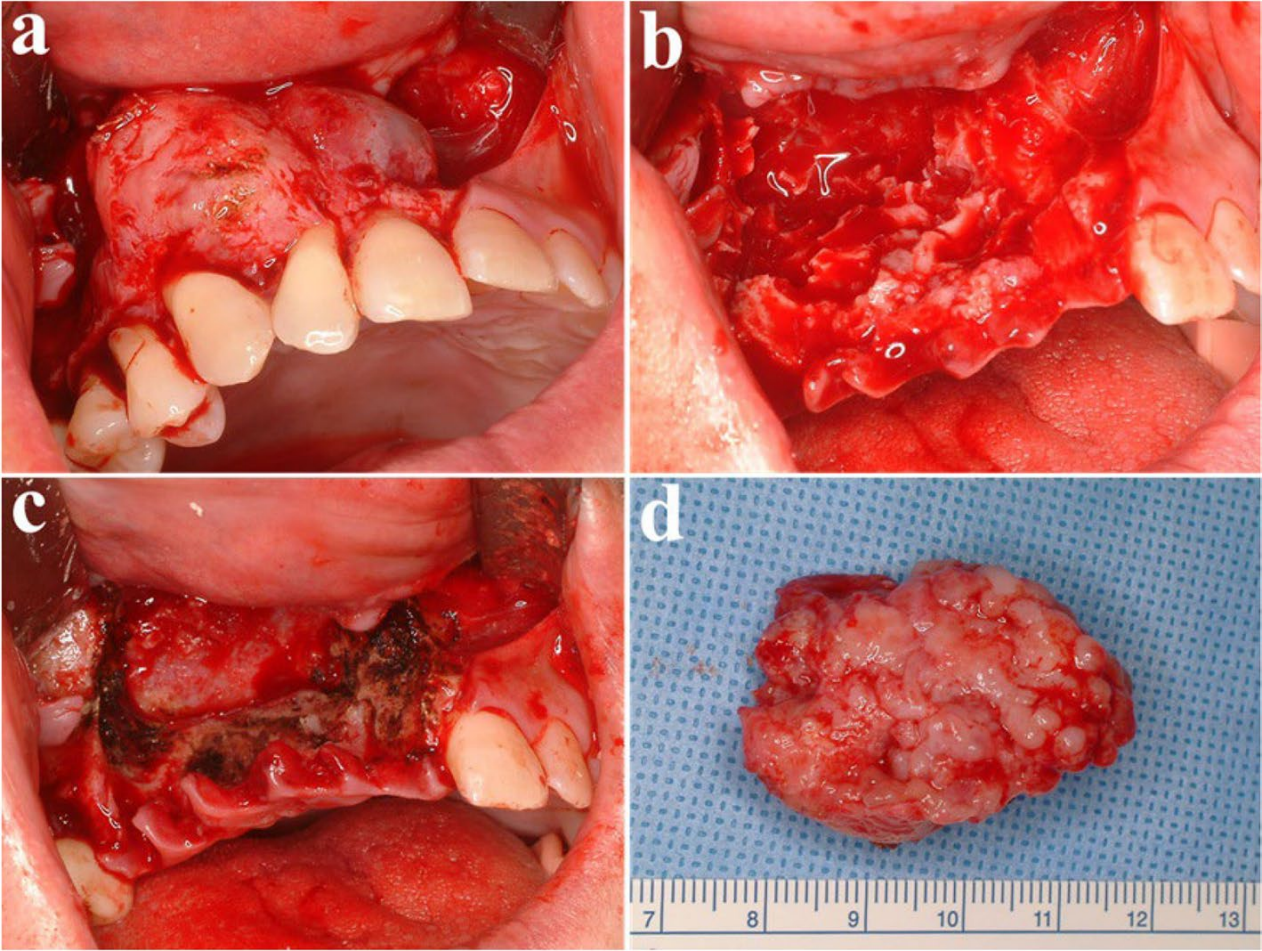
Surgical excision and peripheral ostectomy of ameloblastoma, (**a**) flap elevation, (**b**) tooth extraction and surgical excision of tumor were done, (**c**) peripheral ostectomy was done, (**d**) specimen

**Fig. 27:**
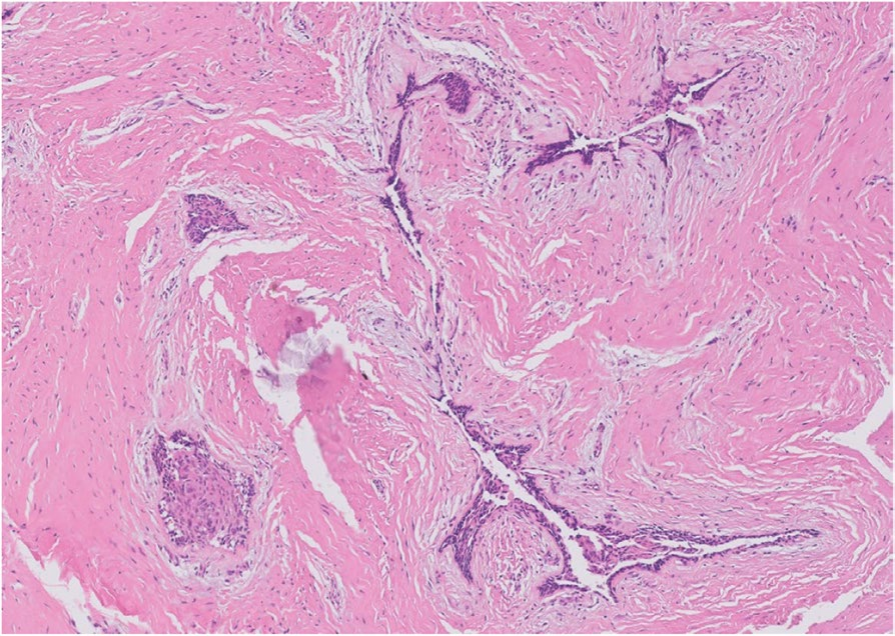
Histopathological slide (× 10), desmoplastic ameloblastoma

**Fig. 28:**
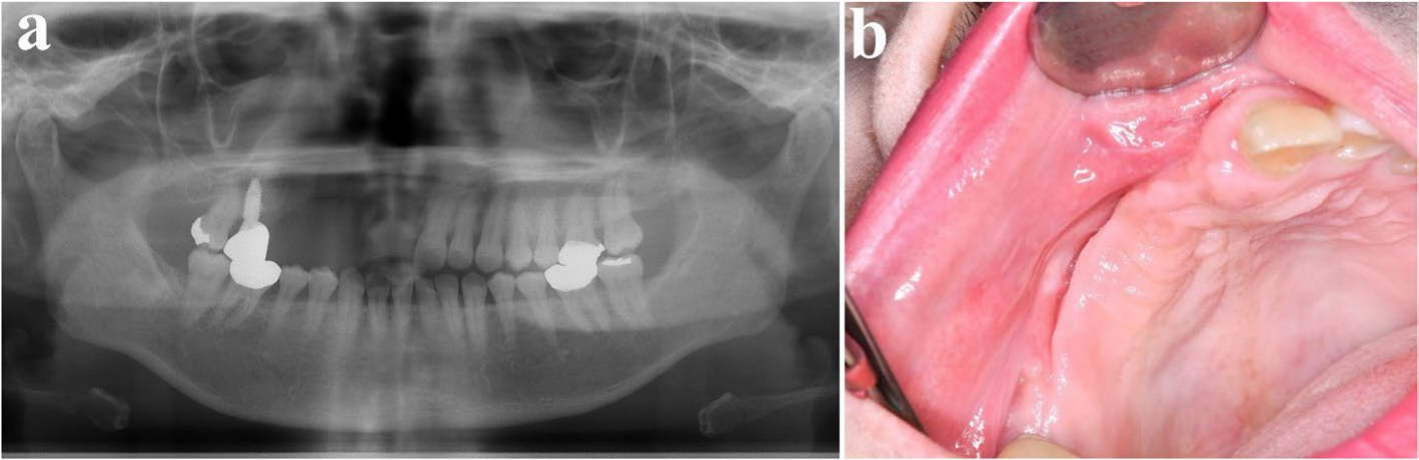
Postoperative radiograph and intraoral photograph **a** Panoramic radiograph at 2 years and 9 months postoperatively shows no evidence of recurrence. **b** Clinical photo at 2 years and 9 months

A 53-year-old female was referred from a local dental clinic for evaluation of a radiolucent lesion on right maxilla (Fig. 25). Her medical history was significant for hypertension. CT imaging revealed a large radiolucent lesion involving multiple teeth, with displacement along the long axes of the affected teeth. Clinical examination showed swelling of the maxillary buccal vestibule. With a provisional diagnosis of ameloblastoma, surgical excision and peripheral ostectomy were done under general anesthesia (Fig. 26 a ~ d). The involved teeth—including the right maxillary central incisor, lateral incisor, canine, first premolar, and second premolar—were extracted. Histopathological examination confirmed the diagnosis of ameloblastoma, with the subtype identified as the desmoplastic type (Fig. 27). The patient has been using a temporary denture, and no recurrence has been observed during more than three years of follow-up (Fig. 28).

## Discussion

### Recurrence rate

In this study, the recurrence rates of OKC (10.5%) and ameloblastoma (0%) were lower than those reported in previous studies, particularly for OKC. A study of OKC analyzing 81 patients with a mean follow-up of 5 years (range: 1–12 years) reported a 14.8% recurrence rate following the surgical excision and peripheral ostectomy [15]. A network meta-analysis of OKC treatment, based on 16 clinical studies involving 711 patients, reported a recurrence rate of 36.7% after surgical excision and peripheral ostectomy [25]. However, there are limited reports on ameloblastoma following surgical excision and peripheral ostectomy. In a previous study of ameloblastoma treated with enucleation and peripheral ostectomy, the recurrence rate was 21.4% (14 patients, mean follow-up of 47 months) [26]. Also, a systematic review analyzing 942 cases reported a 30% recurrence rate for ameloblastoma treated with conservative surgical approaches [27]. Our findings contrast with the traditional preference for more radical surgery in the treatment of ameloblastoma.

In this study, the recurrence rate was higher in OKC (10.5%) compared with ameloblastoma (0%), although this difference did not reach statistical significance (*p* = 0.244).

#### Sinus invasion

OKC predominantly occurred in the maxillary posterior region, often associated with impacted third molars and frequent invasion into the maxillary sinus (Table 2). As a result, the surrounding bone available for peripheral ostectomy was often insufficient, and no additional treatment was applied to the underlying sinus mucosa, potentially allowing for remnant epithelial cells to persist. Additionally, the limited surgical access and poor visibility in the posterior maxilla may have contributed to incomplete removal of the lesion. Notably, all recurrent cases in this study were in the maxillary posterior region with maxillary sinus invasion, further supporting the impact of anatomical challenges on recurrence rates.

Conversely, ameloblastoma in this study was often surrounded by bone, allowing for more extensive peripheral ostectomy and better surgical access, which may have contributed to the absence of recurrence in this group. This difference in surgical accessibility and ability to perform thorough bone removal may explain why OKC demonstrated a higher recurrence rate compared to ameloblastoma despite undergoing the same surgical approach.

#### Consistency

The cyst lining of OKC typically consists of a uniform, narrow layer of keratinized stratified squamous epithelium, measuring approximately 5–8 cell layers in thickness without the presence of rete ridges (Figs. 3 and 13). The keratinization is para-keratinized in about 80–90% of cases [28]. The epithelial lining shows a fairly consistent thickness, averaging around 365.82 μm [29]. Due to the fragile and thin cyst lining epithelium, it was frequently observed that the cyst wall tended to tear during enucleation of OKC. In such cases, remnants of cystic tissue might be left behind. If these remnants are not fully removed during the subsequent peripheral ostectomy, it is suspected that they could contribute to recurrence.

Among ameloblastoma, on the other hand, the conventional type accounts for approximately 91%, while the unicystic and peripheral types account for about 6% and 2%, respectively [30]. Except for the unicystic type, ameloblastoma generally presents as solid mass, which makes them less prone to tearing and easier to remove as a whole. In this study, only one case of ameloblastoma was classified as unicystic, and it was removed without cystic tearing. All other cases presented as solid masses, allowing for en-bloc removal.

#### Surgical sight, accessibility

In all cases of OKC recurrence, the recurrent lesions developed at a site more distal than the original lesion, most commonly around the pterygoid plate area. Surgical access and visualization were particularly challenging in the region posterior to the sinus posterior wall. In addition, no specific management was performed for the underlying mucosa at the base, making it difficult to achieve complete removal of the lesion and suggesting the possibility of residual satellite cysts. Frequent cortical perforation and firm adhesion of the cyst to the overlying mucosa further complicate complete excision, contributing to a higher recurrence rate [31].

One of the recurrent cases involved a patient who had previously undergone two surgeries for OKC at other institutions, 7 and 5 years before our hospital. At the time of presentation, the lesion was located around the pterygoid plate. Removal was performed via Le Fort I osteotomy approach. However, recurrence occurred on the palatal side. It is difficult to determine whether this recurrence was due to residual tissue left after surgery at our hospital or from remnants persisting from surgeries performed 5–7 years earlier. Nevertheless, considering the anatomical location and the difficulty of access, it is possible that incomplete removal may have played a role.

The other recurrent case was also identified in regions more distal than the primary site, near the pterygoid area (Figs. 6 and 9). Even after the second surgery, recurrence was observed in a more distal location (Fig. 9). During the second surgery, a buccal approach was used without performing a Le Fort I osteotomy. Due to the limited surgical field and instrument access, as well as the anatomical complexity of the area, complete and aggressive removal is challenging, which may account for the high likelihood of recurrence.

#### Satellite cyst of OKC

In addition, OKC is characterized histopathologically by the formation of satellite cysts, which contributes to its high recurrence rate (Figs. 3 and 13). Therefore, if satellite cysts remain in the underlying mucosa or bone marrow after cyst enucleation, they may lead to recurrence even after peripheral ostectomy. In contrast, ameloblastoma shows local infiltration and bone destruction within the bone marrow space. Recurrence in ameloblastoma typically occurs following incomplete surgical removal; however, if the extent of the lesion is accurately determined and fully resected, the recurrence rate can be significantly reduced.

#### Impacted molar or conservation of involved tooth

It is well recognized that attempting to preserve teeth involved in the cyst, especially when enucleation alone is performed, often leads to incomplete removal and subsequent recurrence. Whether to extract or preserve teeth involved in the cyst remains a common dilemma in clinical practice. While extraction is clearly indicated for supernumerary, impacted, non-functional, or recurrently involved teeth, the management of other cases remains controversial. Some previous studies suggest that, in any situation where pathological tissue might remain, extraction of the involved teeth should be considered to minimize the risk of recurrence [31].

### Other adjuvant therapy

Various adjunctive therapies after conservative treatment have also been tried to reduce recurrence rates. Among them, cryotherapy and Carnoy’s solution are widely used to improve treatment outcomes. Cryotherapy induces cell death through ice crystal formation as well as electrolyte and osmotic imbalance, effectively eliminating epithelial remnants or satellite cyst cells while preserving the inorganic bone matrix for osteogenesis [32, 33]. Carnoy’s solution consists of absolute alcohol, causing cell dehydration; chloroform, which acts as a lipid solvent enhancing alcohol’s effect but is also a known carcinogen; glacial acetic acid, which has a synergistic effect with alcohol; and ferric chloride, which provides hemostatic properties and stains tissues [34]. Several studies have investigated the impact of adjunctive therapies, such as Carnoy's solution and cryotherapy, on the recurrence rates of OKC and ameloblastoma.

A previous study by Janas-Naze et al. compared the efficacy of Carnoy's solution and modified Carnoy's solution in managing OKC. The study found recurrence rates of 8.2% in the conventional solution group and 10.2% in the modified solution group, indicating comparable effectiveness between the two formulations [35]. In the context of cryotherapy, a systematic review by Sharifian et al. evaluated various adjunctive treatments for OKC. The review concluded that only Carnoy's solution was statistically significant compared to no adjuvant therapy, while there was weak evidence supporting the efficacy of modified Carnoy's solution. The review found no evidence that cryotherapy led to statistically significant improvements over no adjunct therapy [36]. ​

These findings suggest that while adjunctive treatments like Carnoy's solution may reduce recurrence rates, they also carry potential drawbacks such as delayed bone healing, impaired bone regeneration, prolonged surgical time, and a lack of standardized treatment protocols. These issues may diminish the advantages typically associated with conservative surgical approaches [36].

### Rationale for surgical excision and peripheral ostectomy

In this study, surgical excision and peripheral ostectomy, one of conservative surgical approach, was performed. These techniques contributed to minimizing functional and aesthetic impairments while achieving stable clinical outcomes. Even if complete bone regeneration is not achieved after surgery, the overall functional and aesthetic outcomes remain satisfactory, and patients do not perceive any significant aesthetic concerns. In the posterior region, bone defect does not pose significant functional or aesthetic issues. In the anterior region, bone loss could be effectively managed with prosthetic solutions, minimizing any aesthetic deficiencies (Fig. 19).

Another significant finding of this study was the late recurrence of OKC, with the case observed up to 14 years after surgery. It demonstrates the importance of long-term follow-up. Jung HD et al. reported the recurrence rates of OKC; 55.1% recurrence occurred within 3 years after surgery, 74.3% occurred within 5 years, and 94.9% occurred within 10 years, which means 5% of OKC recurrence occurred after 10 years [37]. Ameloblastoma has also been documented to recurrence after as long as 10–15 years from initial treatment, emphasizing the importance of long-term postoperative follow-up for both lesions [38].

In this study, recurrence was observed only in OKC cases; however, the growth rate during follow-up was found to be very slow, with an increase of approximately 1 mm per year (Fig. 9a ~ e). According to previous studies, recurrent OKCs exhibit significant variability in growth rate, averaging approximately 5 mm per year (range: 0.7 ~ 22.0 mm/year), while primary OKCs may grow slightly slower [39]. This suggests that even if recurrence is suspected on postoperative CT, it is possible to differentiate between postoperative changes and recurrent tumors through close observation over time. Moreover, in the event of recurrence during regular follow-up, the reoperation can be performed in a more limited area compared to the primary site, enabling a more conservative surgical approach (Figs. 6 and 9). In contrast, ameloblastoma in the jaw, particularly the solid, multicystic type, has been reported to grow at an average rate of approximately 85 mm per year, with a specific growth rate of nearly 88% annually [40]. This means the tumor can almost double in size within a year if left untreated, with the highest growth rates seen in solid, multicystic subtypes and maxillary lesions. Considering the growth rate upon recurrence, potential for malignant transformation, and recurrence rate, surgical excision and peripheral ostectomy, when combined with careful follow-up, may offer an appropriate risk–benefit balance for both types of tumors and can be considered a primary treatment option.

Although Fisher’s Exact Test did not show a statistically significant difference, the conclusion of this study was derived from a simple comparison of recurrence rates (Table 1). Given the limitations of the sample size, further studies with more cases are necessary to verify these findings. Future research could apply statistical methods such as the Chi-square test or logistic regression analysis to more accurately assess recurrence risk and identify potential contributing factors.

## Conclusion

For the treatment of OKC and ameloblastoma, which are representative benign lesions of odontogenic origin with locally invasiveness and high recurrence rates, surgical excision (or simple enucleation) combined with peripheral ostectomy appears to be a reasonable option when surgical access is feasible and careful long-term follow-up is ensured. However, cases of recurrence have been observed even after long-term follow-up, highlighting the necessity for postoperative monitoring for at least 10 years to ensure comprehensive disease management.

## Data Availability

No datasets were generated or analysed during the current study.

## Abbreviations

OKC: Odontogenic keratocyst
CBCT: Cone beam computed tomography
